# Finger abduction as a novel function of the extensor digitorum brevis manus muscle

**DOI:** 10.1007/s00276-021-02770-1

**Published:** 2021-06-13

**Authors:** Kalpesh R. Vaghela, Craig Brownlie, Dafydd S. Edwards

**Affiliations:** 1 Department of Trauma and Orthopaedic Surgery, Royal London Hospital, Barts Health NHS Trust, London, UK; 2 Queen Mary University of London, London, UK

**Keywords:** Extensor digitorum brevis manus muscle, Finger abduction, Accessory muscle, Extensor digitorum brevis medius muscle

## Abstract

A 25-year-old female presented with a chronic scapho-lunate ligament injury with development of carpal instability requiring reconstruction. During a standard dorsal longitudinal mid-line approach to the carpus, an extensor digitorum brevis manus (EDBM) muscle was found taking its origin from the dorsal wrist capsule overlying the lunate with innervation from the posterior interosseous nerve (PIN). Electrical stimulation of the muscle belly demonstrated abduction of the middle finger. The EDBM is a rare anatomical variant of the extensor compartment of the wrist and may be encountered during surgical approaches. Where possible these variant muscles should be carefully dissected off underlying structures, preserved and repaired at the conclusion of a procedure to ensure no perceived functional deficit to the patient. We present a case of a previously undescribed EDBM muscle function of pure finger abduction with no extension and a surgical technique of preserving its origin. We propose that the middle finger variant of the EDBM should be re-named the extensor digitorum brevis medius to reflect our findings.

**Supplementary Information:**

The online version contains supplementary material available at 10.1007/s00276-021-02770-1.

## Introduction

The extensor digitorum brevis manus (EDBM) muscle is a rare anatomical variant of the extensor compartment of the wrist and hand. It was first described by Bernard Siegfried Albinus in 1758 who named it “Extensor brevis digiti indicis vel medii” [1]. The name EDBM was ascribed to the muscle by Macalister in 1875 which has been widely used by authors in the literature [9]. The EDBM typically originates from the dorsal wrist capsule, the dorsal distal radius, dorsal metacarpal surface or proximal radiocarpal ligament overlying the fourth extensor compartment [4, 14]. It can have up to four tendons with the most commonly occurring pattern being a single tendon to the index or middle finger. Ogura classified the EDBM according to its distal insertion and relationship with extensor indicis proprius (EIP) muscle. Type I: Absent EIP with EDBM attached to index finger dorsal aponeurosis. Type II is where both EDBM and EIP insert onto the index finger. It has three subtypes which describe how they interact. Type IIa: A vestigial EIP is confluent with an EDBM muscle belly and inserted onto the index finger. Type IIb: The distal end of the EDBM muscle belly joins the EIP tendon. Type IIc: EIP inserts normally onto the index finger along with a thin EDBM tendon also inserted more ulnarly than the EIP tendon. Type III: The EIP inserts onto the index finger but the EDBM inserts onto the middle (long) finger ± an accessory EIP to the middle finger [5].

It is innervated by the posterior interosseous nerve (PIN) and takes its blood supply from a posterior branch of the anterior interosseous artery. When the EIP was absent, the EDBM was found to be present in 50% in cadaveric specimens which suggests that it compensates for EIP and supports the notion that EDBM is a finger extensor [16]. In a recent systematic review and meta-analysis, the EDBM muscle has an overall true cadaveric prevalence of 2.5% and a bilateral occurrence in 26.3%. There was no significant association found between EDBM and gender, ancestry or laterality [20].

Variations in intrinsic hand muscle anatomy can be encountered during common surgical approaches such as the dorsal approach to the carpus. We present a case of a previously undescribed EDBM muscle function of pure finger abduction with no extension of the middle finger and a surgical technique of preserving its origin.

## Case report

A 25-year-old right hand dominant radiographer had a fall several years ago and sustained a scapho-lunate ligament injury which was not treated. This resulted in scapho-lunate diastasis and symptomatic instability. The Kirk-Watson test was positive and radiographs of the wrist revealed no evidence of scapho-lunate advanced collapse (SLAC) wrist. A wrist arthroscopy confirmed the scapho-lunate ligament injury. She underwent a scapho-lunate ligament reconstruction using the tri-ligament tenodesis (3LT) technique which is a modification of the Brunelli procedure [17].

Intra-operatively, a standard dorsal longitudinal mid-line approach to the wrist was performed; Listers tubercle was identified and a longitudinal incision was made 4 cm distally and 4 cm proximally to the radiocarpal joint over the 3rd/4th extensor compartment. The extensor retinaculum was exposed and step cut was performed to access the dorsal capsule of the carpus. The extensor pollicis longus (EPL) tendon was liberated and retracted radially and the extensor digitorum longus (EDL) and EIP were retracted ulnarly.

**Fig. 1:**
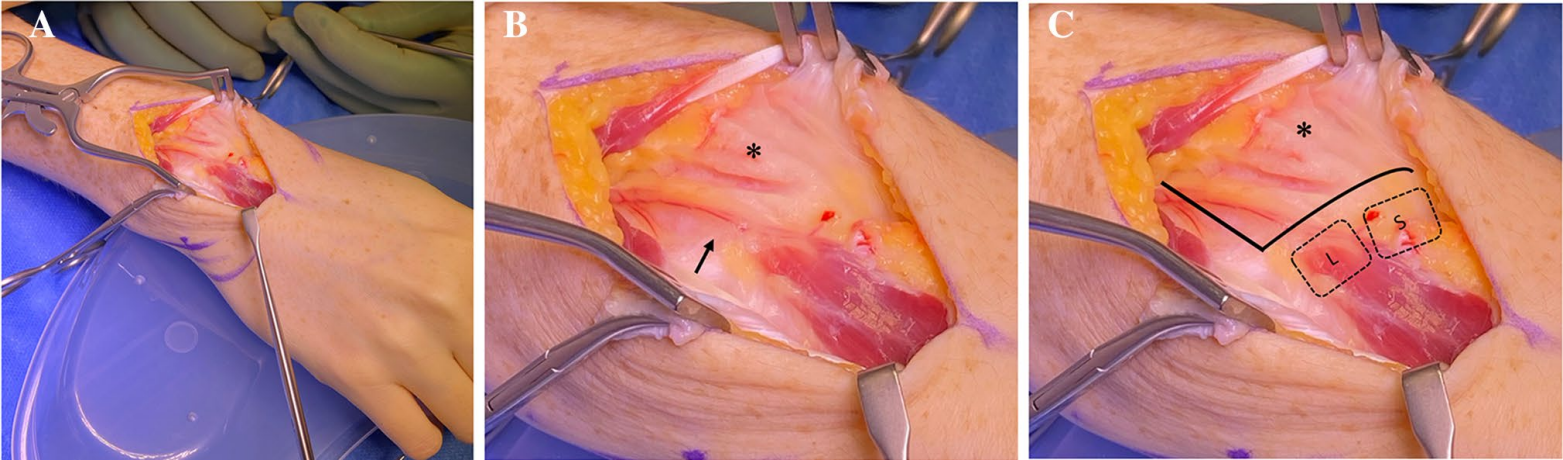
**a** Intraoperative photograph of the wrist. **b** The Listers tubercle is marked with an asterisk (*) and the posterior interosseous nerve (PIN) is marked with an arrow. **c** The boundaries of the distal radius (lines), listers tubercle (asterisk), lunate (L) and scaphoid (S) bones are outlined

An EDBM muscle belly was found to be originating from the dorsal capsule overlying the lunate bone. It was found to originate ulnarly from Listers tubercle just distal to the 4th dorsal compartment of the wrist and inserted onto the ulnar side of the proximal phalanx of the middle finger making it an Ogura Type III EDBM. A branch of the PIN and accompanying vessel were found to be supplying innervation the muscle belly proximally (Fig. 1). Upon electrical stimulation of the EDBM muscle, it was found to abduct the middle finger with no extension of the metacarpophalangeal joint (Video 1).

**Fig. 2:**
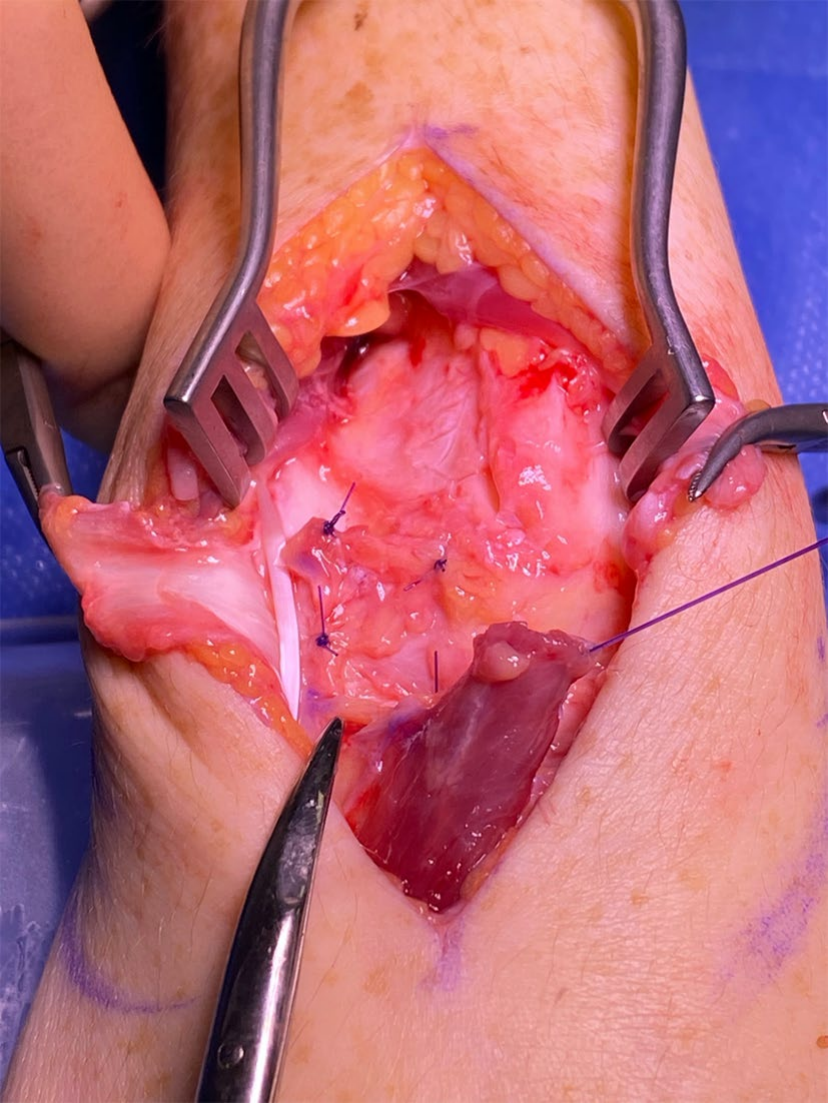
The extensor digitorum brevis manus muscle is reflected off the capsule. The interrupted polyester dioxanone (PDS) sutures have been used to close the Berger flap

To gain access to the proximal row of the carpal bones, the EDBM muscle was reflected off the dorsal capsule using a blade paying careful attention not to damage the neurovascular pedicle. A stay suture was used to reflect the muscle origin and ensure that the muscle was not damaged during the scapho-lunate reconstruction. Stimulation of the muscle confirmed the insertion of the tendon onto the proximal phalanx due to its action (Fig. 2).

A Berger flap is a radially based dorsal capsular flap created by longitudinally splitting the dorsal radiocarpal and the dorsal intercarpal ligaments with the apex at the triquetrum and base at the scaphoid to expose the carpal bones [2]. The flap is radially based to preserve the dorsal blood supply to the scaphoid. A tri-ligament tenodesis was performed to reconstruct the scapho-lunate ligament. At the conclusion of the procedure, the EDBM muscle belly was repaired and was found to be responsive to electrical stimulation.

## Discussion

This case describes a hitherto undescribed function of the EDBM muscle. We found that upon electrical stimulation of the muscle belly the EDBM caused pure middle finger abduction with no extension which is analogous to the function of the dorsal interossei. The EDBM as its name suggests has always been considered a metacarpophalangeal joint extensor as it shares its nerve and blood supply with the EIP.

From an evolutionary and embryological perspective, this function holds true. It is thought that the EDBM is related to extensor brevis which is a primitive intrinsic amphibian extensor. It is thought that in humans, the EDBM is a atavistic muscle and occurs due to failure of the proximal migration of the ulnocarpal elements of the antebrachial muscle mass [10]. Furthermore, in the human embryo, the precursor extensor muscles differentiate into radial, superficial and deep portions the latter of which is innervated by the PIN. The deep portion is further divided into the radial and ulnar sides. On the radial side, it gives rise to the abductor pollicis longus (APL) and the extensor pollicis brevis (EPB) and on the ulnar side EPL and EIP. It has been shown that the deep portion is highly variable and has undergone significant evolutionary changes. The EDBM has likely developed from the deep unstable portion of the forearm extensor muscle mass [15, 19].

There are few studies which investigate the in vivo function of the EDBM. Patel et al. utilised EDBM as a tendon transfer to restore function of a post-traumatic injury to EPL. When the EDBM tendon was “tugged”, it resulted in extension of the index finger metacarpophalangeal joint and was, therefore, used in the transfer. The tendon transfer successfully restored thumb retropulsion [12]. It should be noted that when manually pulling on a tendon, it may cause movement of a joint in a non-physiological direction due to the force vector being applied by the surgeon and not due to contraction of the muscle belly. In our case, we used electrical stimulation to avoid this issue.

To our knowledge, there are four clinical reports that performed electromyography of the EDBM to study its action. Egawa and Hashimoto were the first to demonstrate electromyographically that the action of EDBM was the same as the EIP [5]. Onesti et al. found an EDBM muscle in a patient with type 1 neurofibromatosis and performed electromyography. Needle electromyography of the EBDM muscle during voluntary extension of the index finger showed poor recruitment and large motor unit potentials with frequent cramp discharges in the EDBM muscle [11]. Reef et al. also found EDBM muscle potentials on finger extension [13]. All three of these studies focused on finger extension only and did not examine finger abduction. The sole paper to describe finger deviation as a function of EDBM was Gama in 1983. Gama stated that “when electrically stimulated the EDBM deviates the proximal phalanx to the side on which it is inserted in relation to the extensor digitorum communis” [7].

The EDBM is a clinically relevant entity and can be the cause of swellings or chronic pain of the dorsum of the wrist. The EDBM can be associated with dorsal wrist ganglia and can pose a diagnostic challenge [3, 6]. Hayashi et al. coined the term Fourth Compartment Syndrome where the EDBM is one of its causes [8]. When present and symptomatic, the EDBM muscle can be released by division of the extensor retinaculum or ablated completely [18].

Surgeons should be cognizant of the variant musculature of the dorsum of the wrist. In addition to the EDBM, other muscles such as the extensor medii proprius (EMP), extensor indicis et medii communis (EIMC) and extensor pollicis et indicis (EPI) may be encountered which may confuse the operating surgeon and inadvertently lead to damage or use as a donor in tendon transfers.

Variant muscles including the EDBM with their neurovascular supply can be injured when performing the dorsal approach to the carpus, during internal fixation of carpal bones or metacarpal fractures or as in this case reconstruction of intrinsic carpal ligaments. Careful dissection of the neurovascular bundle supplying the muscle should be performed and the muscle should be reflected off capsule and bone in a single layer. A stay suture can be helpful in preventing fraying and damage to the muscle when being retracted to gain surgical access. We recommend repair of the muscle with absorbable monofilament sutures.

There are reports of ulnar-sided EDBM variations which do not fit with the Ogura classification which is centered around the EIP and radial side of the hand. In a cadaveric study, Cavdar et al. found a band shaped EDBM located between the extensor tendons of the ring and little fingers lying superficial to the interosseous muscles. The tendon inserted on the ulnar side of the extensor tendon and the dorsal digital expansion of the ring and little fingers and was supplied by a fine branch of the PIN. The EIP and extensor digiti minimi (EDM) were both present [3]. It stands to reason that the ulnar-sided insertion of the EDBM in this study would cause finger abduction, however, this hypothesis was not tested due to its cadaveric nature.

This case demonstrates that the EDBM muscle has novel functions such as finger abduction which has not been previously described and suggests that variations of the EDBM may be more complex than once thought. We propose that the middle finger variant (Ogura Type III) of the EDBM should be re-named the extensor digitorum brevis medius to reflect our novel in-vivo findings.

## Supplementary Information

Below is the link to the electronic supplementary material.

Supplementary file1 (MOV 8088 kb) Video 1 – Intraoperative video of electrical stimulation of the Extensor Digitorum Brevis Manus muscle causing abduction of the middle finger with no active extension
